# Axillary Aneurism; Absence of Diagnostic Signs; Ligation of the Left Subclavian Artery

**Published:** 1870-10

**Authors:** C. C. F. Gay

**Affiliations:** Surgeon to the Buffalo General Hospital


ART. III.—*Axillary Aneurism; Absence of diagnostic signs; Ligation of the left subclavian artery. Recovery.* By C. C. F. GAY, M. D., *Surgeon to the Buffalo General Hospital.*

George Saltzman, aged 26 years, was admitted into the surgical ward of the Hospital on August 26th, 1870. He was wounded in 1864 by the accidental discharge of a pistol, the ball entering the front of the left shoulder. Search was made for the ball in vain. Immediately after the accident a small tumor was observed in the axilla. The fore-arm and arm became partially paralyzed. For the past six or eight months the tumor has been growing very rapidly, until it has now attained to nearly the size of a child's head. The tumor occupies a position just anterior to the axilla, or at the sub-clavicular region, feels hard, is movable, and lately has become pointed like an abscess about to break, feels soft, and fluctuates over a space one inch and a half in diameter. There is no pulsation, either in the tumor or at the wrist. On applying the stethescope no thrill is detectible; neither is the size of the tumor diminished by pressure.

The patient has consulted many surgeons and physicians of this city, and also of Detroit, and been advised—on account of the uncertainty in diagnosis—to let the tumor alone; but latterly it has become so troublesome he has entered the Hospital with the determination to have something done, regardless of consequences. Accordingly, I invited my colleagues, both of the surgical and medical staff, to meet me on Aug. 30th. As no one present was able to say, unequivocally, that the case was one of aneurism after having resort to all the known means of diagnosis, I introduced the exploring trochar into the tumor, drawing off a small quantity of thick and blood-colored fluid; carrying the point of the trochar into the middle of the tumor no fluid escaped. Next I introduced at the same point an ordinary trochar and obtained more fluid, but as there was so little of it, and it had ceased to run, it was concluded that the fluid that escaped occupied the superficial portion of the tumor, which fluctuated to the touch. On consultation it was deemed advisable to make an exploratory incision over the tumor, and if it could be ascertained that it was other than aneurismal, to remove it. Accordingly chloroform was administered, when I made, with great caution, an incision about eight inches in length—over the tumor—and came down upon a bluish-colored surface, upon which I made gentle pressure with the handle of the scalpel when the aneurismal sac burst. Instantly I tore open the sac in the line of incision made, turned out the coagulum, seized the axillary vessels, suppressing the hemorrhage and holding on firmly until the subclavian could be compressed, then withdrawing the hand I assigned this position to two asssistants and proceeded at once to ligate the subclavian artery. The operation lasted one hour, and was interrupted and prolonged by the necessity of administering stimulus on the approach of syncope. Ether was then given in the place of chloroform and the operation completed.

The artery was ligated at the junction of the second and third portion of its course. Considerable hemorrhage followed the operation, proceeding apparently from the surface of the aneurismal walls, necessitating the plugging with sponges dipped in Sol. Ferri Persulphas. The patient was placed in bed and whisky again given with an anodyne.

On the night of September 5th, secondary hemorrhage supervened, but was at once arrested by the house physician and his assistants.

The axillary wound was firmly tamponed with sponge, wet with the solution of iron, and the plug allowed to remain for three days, on the removal of which there was not so much as any oozing, even, of blood from the wound. The ligature came away spontaneously on the 17th day after the operation.

On October 11th, six weeks from the date of the operation, the patient is up and dressed, having a good appetite and feels well. The wound is closed, but there remains a partial paralysis, and there is still no pulsation in the radial artery.

My obligations are due to my colleagues and other medical gentlemen present for their assistance, cheerfully and ably rendered; and especially due to the assistance rendered to the patient at night by the house physician, Dr. Kitchel, and the assistant house physician, Mr. Harrington, medical student, without which my patient must have perished from secondary hemorrhage.

